# Clinical Practice Guidelines for Pancreatic Cancer 2022 from the Japan Pancreas Society: a synopsis

**DOI:** 10.1007/s10147-023-02317-x

**Published:** 2023-03-15

**Authors:** Takuji Okusaka, Masafumi Nakamura, Masahiro Yoshida, Masayuki Kitano, Yoshinori Ito, Nobumasa Mizuno, Keiji Hanada, Masato Ozaka, Chigusa Morizane, Yoshifumi Takeyama, the Committee for Revision of Clinical Guidelines for Pancreatic Cancer of the Japan Pancreas Society, Yasunari Sakamoto, Dai Inoue, Ken Kamata, Manabu Kawai, Atsushi Kanno, Masahiro Serikawa, Atsushi Sofuni, Yukiko Takayama, Hiroyuki Matsubayashi, Takao Ohtsuka, Shunsuke Onoe, Manabu Kawai, Masayuki Sho, Tsutomu Fujii, Ippei Matsumoto, Ryusei Matsuyama, Masamichi Mizuma, Tomohisa Yamamoto, Tatsuya Ioka, Hidetoshi Eguchi, Masanori Someya, Kohei Nakata, Satoaki Nakamura, Takayuki Ohguri, Makoto Shinoto, Masanori Someya, Satoaki Nakamura, Tatsuya Ioka, Makoto Ueno, Takao Itoi, Hiroyuki Isayama, Hironari Kato, Yousuke Nakai, Keiichi Uemura, Takuya Oyakawa, Hatoe Sakamoto, Yoichi Shimizu, Keita Tagami, Tetsuya Tsuji, Maiko Fujimori, Shuichi Mitsunaga, Masanori Mori, Takashi Yokokawa, Masashi Kanai, Hiroyuki Matsubayashi, Shoji Osada, Yoichi Shimizu, Sawako Furutani, Yoshiyuki Majima, Masanori Mori, Naohiko Yamaguchi, Natsuki Narita, Akiko Okumura, Kyoko Shima, Yasunari Sakamoto, Keiko Kondo, Nobuya Akizuki, Reiko Ashida, Naoki Ikenaga, Yasutaka Ishii, Shigeto Ishii, Noboru Ideno, Koji Inaba, Yu Uneno, Rei Umezawa, Katsuhisa Ohgi, Akihiro Ohba, Shunsuke Omoto, Yoshiro Okajima, Yuka Ono, Akiyoshi Kasuga, Keiko Kamei, Hiroshi Kurahara, Hidekazu Kuramochi, Keisuke Kurihara, Takamichi Kuwahara, Kazuo Kobayashi, Satoshi Kobayashi, Tomohiro Kondo, Kei Saito, Taro Shiga, Taro Shibuki, Kazuto Shibuya, Akinori Shimizu, Yasuhiro Shimizu, Hidenori Takahashi, Naminatsu Takahara, Takaaki Tsuchiya, Takayoshi Tsuchiya, Tomofumi Tsuboi, Fumihito Toshima, Ko Tomishima, Hiroki Nagai, Kenji Nakagawa, Tsuyoshi Hamada, Satomi Higashigawa, Yuji Higuchi, Yusuke Hiratsuka, Toshio Fujisawa, Hideki Funahashi, Kazuyuki Matsumoto, Takayuki Miura, Takafumi Mie, Kosuke Minaga, Kentaro Miyake, Motoki Miyazawa, Tetsurou Miwata, Kaori Muraoka, Yasuhiro Yabushita, So Yamaki, Tomohiro Yamazaki, Yasunobu Yamashita, Kensuke Yamada, Kenjiro Yamamoto, Kensuke Yokoyama, Yukihiro Yokoyama, Isaku Yoshioka, Koji Yamaguchi, Kyoko Shimizu, Masanori Sugiyama, Toshio Nakagohri, Makoto Nakamuta, Yosuke Hatakeyama, Junji Furuse, Fuyuhiko Motoi, Kenji Yamao, Masato Watanabe, Eiji Ishikawa, Atsuko Kitano, Nobumasa Takagaki, Hironobu Tokumasu, Hiroshi Noto

**Affiliations:** 1 Department of Hepatobiliary and Pancreatic Oncology, National Cancer Center Hospital, 5-1-1 Tsukiji, Chuo-ku, Tokyo, 104-0045 Japan; 2 Department of Surgery and Oncology, Graduate School of Medical Sciences, Kyushu University, Fukuoka, Japan; 3 Department of Hepato-Biliary-Pancreatic and GI Surgery, International University of Health and Welfare, Ichikawa, Japan; 4 Second Department of Internal Medicine, Wakayama Medical University, Wakayama, Japan; 5 Department of Radiation Oncology, Showa University School of Medicine, Tokyo, Japan; 6 Department of Gastroenterology, Aichi Cancer Center Hospital, Nagoya, Japan; 7 Department of Gastroenterology, JA Onomichi General Hospital, Onomichi, Japan; 8 Hepato-Biliary-Pancreatic Medicine Department, Cancer Institute Hospital, Tokyo, Japan; 9 Department of Surgery, Kindai University Faculty of Medicine, Osaka, Japan

**Keywords:** Pancreatic cancer, Clinical guidelines, Japan Pancreas Society, Minds, GRADE system

## Abstract

**Objectives:**

Clinical Practice Guidelines for Pancreatic Cancer was first published in 2006 by the Japan Pancreas Society, and revised in 2009, 2013, 2016, and 2019. In July 2022, Clinical Practice Guidelines for Pancreatic Cancer was newly revised in Japanese.

**Methods:**

For this revision, we developed an entirely new guideline according to the Minds Manual for Guideline Development 2020, which includes the concepts of GRADE—Grading Recommendations Assessment, Development, and Evaluation, to enable a better understanding of the current guidelines. Patients and the public were actively involved in both the development and implementation of the guideline.

**Results:**

The guideline includes algorithms for diagnosis, treatment, chemotherapy, and precision medicine of pancreatic cancer, and addresses 7 subjects: diagnosis, surgical therapy, adjuvant therapy, radiation therapy, chemotherapy, stent therapy, and supportive & palliative medical care. It includes 73 clinical questions and 112 statements. The statements correspond to the clinical questions, evidence levels, recommendation strengths, and agreement rates.

**Conclusions:**

This guideline represents the most standard clinical and practical management guideline available until date in Japan. This is the English synopsis of the Clinical Practice Guidelines for Pancreatic Cancer 2022 in Japanese, and is an attempt to disseminate the Japanese guideline worldwide to introduce the Japanese approach to the clinical management of pancreatic cancer.

## Introduction

The Clinical Practice Guidelines for Pancreatic Cancer based on Evidence-Based Medicine 2006 [1] was first published by the Japan Pancreas Society (JPS), and has undergone repeated revisions: in July 2009 [2, 3], October 2013 [4, 5], October 2016 [6, 7], and July 2019 [8, 9], with the last new revision published in July 2022 [10]. For this latest revision, we developed an entirely new guidelines according to the Minds Manual for Guideline Development 2020 [11]. There were changes in the composition of the committee members for this revision, and more specialists from a wide variety of fields were included to avoid biases in the recommendations. This guideline represents the most standard guideline for the clinical and practical management of pancreatic cancer available until date in Japan. We prepared this English synopsis of the Clinical Practice Guidelines for Pancreatic Cancer 2022 in Japanese, in an attempt to disseminate the Japanese guidelines worldwide, to introduce the Japanese approach to the clinical management of pancreatic cancer.

## General outline of the revision process

**Fig. 1:**
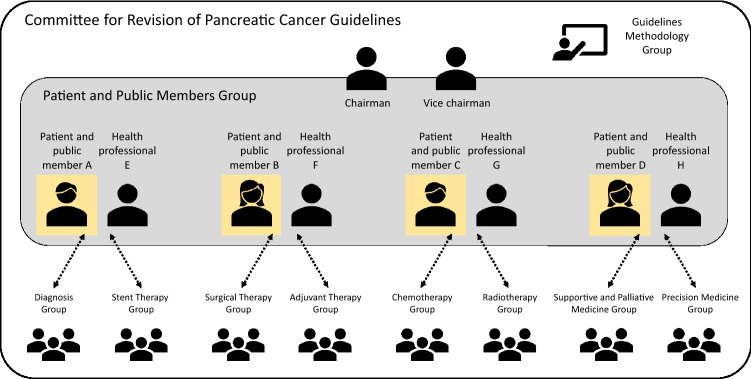
Organizational structure for patient and public involvement in the development of the pancreatic cancer guideline

The composition of the committee for Revision of the Clinical Guidelines for Pancreatic Cancer in the JPS was as follows: Takuji Okusaka, Chairman; Masafumi Nakamura, Vice-Chairman; Masahiro Yoshida, Masayuki Kitano, Yoshinori Ito, Nobumasa Mizuno, Keiji Hanada, Masato Ozaka, and Chigusa Morizane, chiefs of the various groups; and 42 other specialists (medical doctors specialized in internal medicine, surgery, gastroenterology, medical oncology, radiology, endoscopy, psycho-oncology, nutrition, palliative & supportive medicine, a nurse specialized in cancer therapeutics, a cancer pharmacist, and medical social workers), 4 representatives of patients and/or members of the public were members of the committee for revision of the guidelines (Fig. 1). In addition, there were 60 other specialists as assistants, 2 advisors from Minds, and 2 librarians who helped with the revision. The revision process with these committee members was begun in July 2020.

**Fig. 2:**
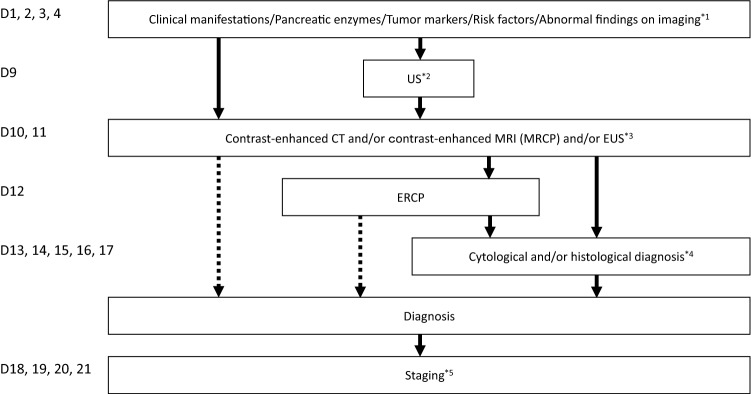
Algorithm for the diagnosis of pancreatic cancer. ERCP: endoscopic retrograde cholangiopancreatography; EUS: endoscopic ultrasonography; MRI: magnetic resonance imaging; MRCP: magnetic resonance cholangiopancreatography; US: ultrasonography; ^*1^Findings during health checkups, comprehensive medical examinations including ultrasonography, screening, and follow-up for other diseases. ^*2^Note that the interpretations depend on proficiency of the attending technician and there is a limit to examining the entire pancreas. If other valuable diagnostic imagings are performed, it may be skipped. ^*3^It is desirable for EUS to be performed at an institution where a high skill level for EUS is available. ^*4^The diagnosis must be established, as much as possible, by histopathology. ^*5^Dynamic CT, dynamic MRI, EUS, PET, and/or laparoscopic examination should be performed as needed

**Fig. 3:**
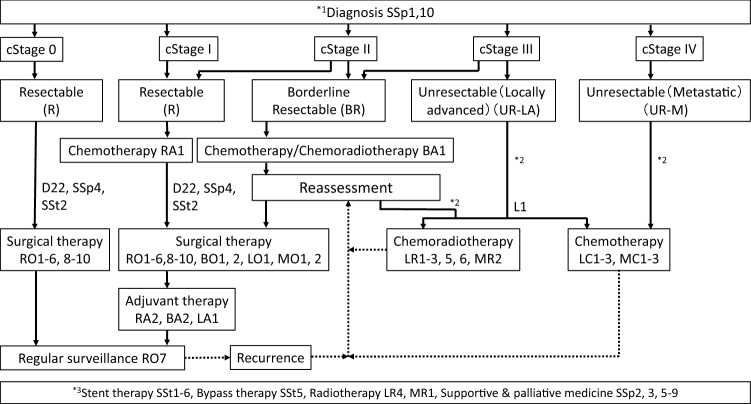
Algorithm for the treatment of pancreatic cancer. Cancer stage classification and classification of the resectability are based on the General Rules for the Study of Pancreatic Cancer, Seventh Edition, Revised and Enlarged Version, the Japan Pancreas Society [12]. *^1^Supportive care for pain, digestion and absorption disorders, pancreatic diabetes, and anxiety are required even from the early stages after diagnosis in patients with pancreatic cancer. For further details, please refer to the guidelines or the HP of the Japanese Society for Palliative Medicine (http://www.jspm.ne.jp/guidelines/index.html). *^2^Please refer to the algorithm for precision medicine. *^3^Stent therapy, bypass therapy, radiotherapy, supportive & palliative medicine, and/or surgical therapy are recommended according to individual patients’ conditions.

**Fig. 4:**
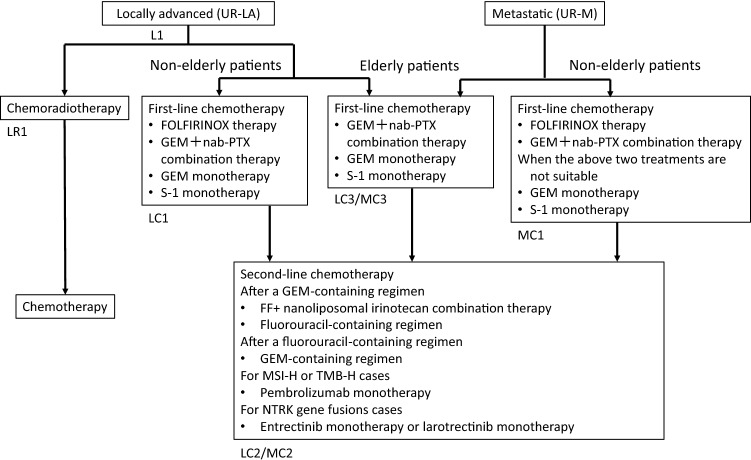
Algorithm for chemotherapy of pancreatic cancer. GEM: gemcitabine; nab-PTX: nab-paclitaxel; FF: fluorouracil + calcium folinate; MSI-H: microsatellite instability-high; TMB-H: tumor mutational burden-high

**Fig. 5:**
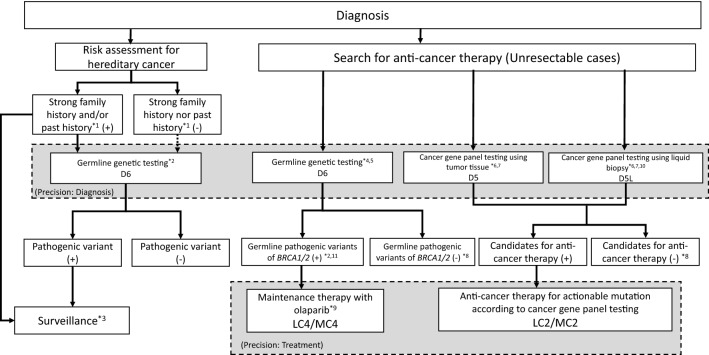
Algorithm for precision medicine of pancreatic cancer. ^*1^Please refer to D4, ^*2^ Genetic counseling is recommended. ^*3^Surveillance to identify hereditary cancer is recommended for blood relatives of patients with pancreatic cancer, Surveillance to identify cancer(s) other than pancreatic cancer is proposed for patients with pancreatic cancer, ^*4^Companion diagnostics (BRACAnalysis®), ^*5^This testing before systemic chemotherapy is covered by health insurance in Japan, ^*6^This testing is covered by health insurance in Japan after standard chemotherapy, ^*7^Genetic counseling is recommended for patients who test positive for a pathogenic germline variant or somatic mutation suspected pathogenic germline variant, ^*8^Standard chemotherapy is recommended independently of genomic findings, ^*9^Maintenance therapy with olaparib is recommended for patients whose disease has not progressed for a certain period of time on a platinum-containing chemotherapy regimen, ^*10^Cancer gene panel testing using blood samples is covered by health insurance in Japan if tumor tissue testing is not feasible or fails, ^*11^ ‘POSITIVE FOR A DELETERIOUS MUTATION’ and ‘GENETIC VARIANT, SUSPECTED DELETERIOUS’ according to BRACAnalysis®

The committee members proposed the guideline, which includes algorithms for the diagnosis (Fig. 2), treatment (Fig. 3), chemotherapy (Fig. 4), and precision medicine (Fig. 5) of pancreatic cancer, and the general consensus for uncontroversial “background questions” and well-established recommendations for diagnosis, surgical therapy, adjuvant therapy, radiotherapy, chemotherapy, stent therapy, and supportive & palliative therapy of pancreatic cancer; there are also particular discussions consisting of “clinical questions (CQs)” and recommendations. A comprehensive search of the literature for the latest articles published after January 1990 (the year in which the literature search was performed for the first version of the guidelines) was performed for each CQ by two librarians (Mr. Naohiko Yamaguchi and Ms. Natsuki Narita). A total of 1,226 articles were collected from 17,769 reports pertaining to pancreatic cancer that were listed on PubMed and Igaku Chuo Zasshi (ICHUSHI), a Japanese bibliographic database, from January 1990 to October 2020. The guidelines address particular discussions, including on the following 6 topics pertaining to the diagnosis of pancreatic cancer (22 CQs and 24 statements), treatment of resectable disease (12 CQs and 16 statements), treatment of borderline resectable disease (4 CQs and 7 statements), treatment of locally advanced disease (13 CQs and 28 statements), treatment of metastatic disease (8 CQs and 25 statements), and supportive & palliative medicine (16 CQs and 24 statements). The corresponding CQ numbers are inserted in the algorithms. There are statements pertaining to the CQs, along with the evidence levels, recommendation strengths, and agreement rates.

We used the Minds manual, following the GRADE system approach. The overall quality of the body of evidence across gross studies for each important outcome was assessed. The evidence level was graded on a scale of A (strongest) to D (least strong). Each committee member specialized in the topic of each CQ prepared a draft of the statement, the evidence level, and the recommended strength. Committee members added some references from their own searches and performed meta-analyses independently, as necessary. These were then reviewed, modified, and finalized by all the committee members. The recommended strength was decided considering 4 factors: evidence level, balance of benefits and harms/burdens, patient preferences, and cost–benefits. Finally, the recommendation strengths were divided into 5 categories (1 = strong, recommend adoption on the approach; 2 = weak, propose adoption on the approach; 3 = weak, propose not to adopt the approach; 4 = strong, recommend against adoption of the approach; 5 = ‘no recommendation’ consensus of the attending committee members when at least 67% of the members attended). When the total agreement rate with recommendation of adopting or not adopting the approach was 50% or more and the total rate of agreement with the opposite view was less than 20%, the recommendation supported by 50% or more members was accepted by the committee. When the agreement rate for adopting or not adopting the approach was 70% or more, the strong recommendation was accepted by the committee members. When the agreement rate for “no recommendation” was 50% or more and that for each of the other recommendations was less than 20%, the “no recommendation” was accepted by the committee. If the voting results did not fulfill any of the above-mentioned criteria, the committee members held discussions again and a final voting was held. Non-fulfillment of the above criteria in the final voting led to the decision of “no recommendation”. Acceptability was determined by voting using an online voting system by the committee members who attended.

Patients and the public have been actively involved in both the guideline development and implementation (Fig. 1). For this revision, we have newly organized ‘the patient and public group,’ including four representatives for patients and/or members of the public and four health professionals. Among the four patients and members from the public, two were members of a patient organization for pancreatic cancer, one was that of a patient organization for another cancer, and one was a medical journalist. Four health professionals supported the patients and members from the public to overcome barriers and facilitate effective patient and public engagement and participate in the revision of the guidelines as committee members. To formulate clinical questions and develop recommendations from the points of view of the patients and public, the ‘survey team’ consisting of one patient, one member of the public, and one health professional conducted an online self-administered questionnaire survey, and the ‘systematic review of literature team’ consisting of two health professionals performed a systematic review of the literatures. Although we could not establish recommendations in this process, we discussed the current situations and perspectives regarding the clinical questions in the guidelines. Each member of the patient and public group participated in both meetings for all committee members and smaller meetings for each category, and provided opinions from the points of view of patients and the public to develop recommendations. The patient and public group played a central role in the latest revision of the Clinical Practice Guidelines for Pancreatic Cancer 2022.

To improve and confirm the validity of the guidelines, they were released in a draft form on the JPS website, inviting comments from the public. Simultaneously, they were reviewed by two external appraisal committees independently using the AGREE Reporting Checklists: a group assigned by the JPS that consisted of surgeons (Koji Yamaguchi, Masato Watanabe, Fuyuhiko Motoi, Toshio Nakagohri, and Masanori Sugiyama), gastroenterologists (Makoto Nakamuta, Kenji Yamao, and Kyoko Shimizu), a medical oncologist (Junji Furuse), an epidemiologist (Yosuke Hatakeyama), and two patient representatives with pancreatic cancer, and another group assigned by Minds, including specialists in guidelines methodology (Eiji Ishikawa, Atsuko Kitano, Nobumasa Takagaki, Hironobu Tokumasu, and Hiroshi Noto), independent of the revision committee members. Finally, taking into account the comments from the public and the external reviewers, the guidelines were reviewed and modified again by the revision committee members and finalized. These new *Clinical Guidelines for Pancreatic Cancer 2022* [8] are in compliance with the new *General Rules for the Study of Pancreatic Cancer* published by the JPS in September 2020[12].

## Notes on the use of the guidelines

These guidelines represent the most standard guidelines for clinical and practical care of patients with pancreatic cancer available at this time. However, they should not be used inflexibly for the practical management of individual patients. The JPS is responsible for the statements in these guidelines. The JPS and the committee members are, however, not liable for any consequences arising from any treatment, for which individual physicians involved in the treatment are responsible.

## Algorithms

The algorithms present the flows for the diagnosis, treatment, chemotherapy, and precision medicine of pancreatic cancer. For a detailed explanation of each CQ, please refer to the indicated box (Figs. 2–5).

### Diagnosis or D

D1 Are further investigations recommended, considering the possibility of pancreatic cancer in patients with new-onset diabetes and exacerbation of diabetes?

*Statement:*

Further investigations are recommended in patients with new-onset diabetes and diabetes exacerbation, considering the possibility of pancreatic cancer.

Recommendation strength: weak; evidence level: C; agreement rates (*N = *51): 1 = 0%, 2 = 98%, 3 = 0%, 4 = 0%, and 5 = 2%*.

*N = number of voters; 1 = strong, recommend adoption of the approach; 2 = propose adoption of the approach; 3 = propose not to adopt the approach; 4 = strong, recommend against adoption of the approach; 5 = no recommendation).

D2 Are monitoring and follow-up recommended, in view of the possible development of pancreatic cancer, in patients with chronic pancreatitis?

*Statement:*

Follow-up observation is recommended in consideration of the possible development of pancreatic cancer in patients with chronic pancreatitis.

Recommendation strength: weak; evidence level: C; agreement rates (*N = *51): 1 = 0%, 2 = 100%, 3 = 0%, 4 = 0%, and 5 = 0%.

D3 In patients with intraductal papillary mucinous neoplasms, are scrutiny and follow-up recommended, in consideration of the possibility of concomitant pancreatic cancer?

*Statement:*

Scrutiny and follow-up observation are recommended in view of the possibility of concomitant pancreatic cancer in patients with intraductal papillary mucinous neoplasms.

Recommendation strength: weak; evidence level: C; agreement rates (*N = *49): 1 = 4%, 2 = 96%, 3 = 0%, 4 = 0%, and 5 = 0%.

D4 Is genetic testing recommended for individuals who have not yet developed the disease, but have a suspected hereditary risk of development of pancreatic cancer based on the family history, medical history, etc.?

*Statement:*

Genetic testing is recommended for individuals who have not yet developed the disease, but have a suspected hereditary risk of development of pancreatic cancer based on the family history, medical history, etc.

Recommendation strength: weak; evidence level: C; agreement rates (*N = *51): 1 = 0%, 2 = 98%, 3 = 0%, 4 = 0%, and 5 = 2%.

D5 Is cancer gene panel testing using tumor tissue recommended in patients with unresectable pancreatic cancer?

*Statement:*

Cancer gene panel testing using tumor tissue is recommended in patients with unresectable pancreatic cancer.

Recommendation strength: weak; evidence level: C; agreement rates (*N = *48): 1 = 0%, 2 = 100%, 3 = 0%, 4 = 0%, and 5 = 0%.

D5L (L: liquid biopsy) Is cancer gene panel testing using liquid biopsy recommended in patients with unresectable pancreatic cancer?

*Statement:*

Cancer gene panel testing using liquid biopsy is recommended in patients with unresectable pancreatic cancer.

Recommendation strength: weak; evidence level: C; agreement rates (*N = *48): 1 = 0%, 2 = 96%, 3 = 0%, 4 = 0%, and 5 = 4%.

D6 Is germline genetic testing recommended for patients with pancreatic cancer and their blood relatives?

*Statement:*

1. Germline genetic testing (still not approved for health insurance coverage for pancreatic cancer) is recommended for the purpose of further assessing the risk of carcinogenesis in patients with pancreatic cancer and their relatives.

Recommendation strength: weak; evidence level: C; agreement rates (*N = *47): 1 = 0%, 2 = 98%, 3 = 2%, 4 = 0%, and 5 = 0%.

2. Genetic testing for germline BRCA1/2 is recommended as a companion diagnostic test for olaparib in patients with locally advanced pancreatic cancer.

Recommendation strength: weak; evidence level: C; agreement rates (*N = *47): 1 = 2%, 2 = 96%, 3 = 0%, 4 = 0%, and 5 = 2%.

3. Genetic testing for germline BRCA1/2 is recommended as a companion diagnostic test for olaparib in pancreatic cancer patients with distant metastases.

Recommendation strength: weak; evidence level: C; agreement rates (*N = *47): 1 = 2%, 2 = 98%, 3 = 0%, 4 = 0%, and 5 = 0%.

D7 Is genetic consultation recommended for pancreatic cancer patients found to have germline pathogenic variants or presumed germline pathogenic variant disclosed by, for example, cancer gene panel testing?

*Statement:*

Genetic consultation is recommended.

Recommendation strength: weak; evidence level: C; agreement rates (*N = *49): 1 = 2%, 2 = 98%, 3 = 0%, 4 = 0%, and 5 = 0%.

D8 Is genetic consultation recommended for blood relatives of pancreatic cancer patients found to have germline pathogenic variants or presumed germline pathogenic variant disclosed by, for example, cancer gene panel testing?

*Statement:*

Genetic consultation is recommended.

Recommendation strength: weak; evidence level: C; agreement rates (*N = *49): 1 = 2%, 2 = 98%, 3 = 0%, 4 = 0%, and 5 = 0%.

D9 Is transabdominal ultrasonography recommended as a first-step diagnostic method in patients with suspected pancreatic cancer?

*Statement:*

Transabdominal ultrasonography is recommended as a first-step diagnostic tool in patients with suspected pancreatic cancer.

Recommendation strength: weak; evidence level: B; agreement rates (*N = *51): 1 = 4%, 2 = 96%, 3 = 0%, 4 = 0%, and 5 = 0%.

D10 Is abdominal magnetic resonance imaging (MRI) recommended as a diagnostic tool in patients with suspected pancreatic cancer?

*Statement:*

Abdominal MRI is recommended as a diagnostic tool in patients with suspected pancreatic cancer.

Recommendation strength: weak; evidence level: C; agreement rates (*N = *51): 1 = 2%, 2 = 96%, 3 = 0%, 4 = 0%, and 5 = 2%.

D11 Is endoscopic ultrasonography (EUS) recommended as a diagnostic tool in patients with suspected pancreatic cancer?

*Statement:*

Endoscopic ultrasonography is recommended as a diagnostic tool in patients with suspected pancreatic cancer, especially as it is more sensitive than other imaging modalities for the diagnosis of pancreatic cancer.

Recommendation strength: weak; evidence level: B; agreement rates (*N = *50): 1 = 4%, 2 = 96%, 3 = 0%, 4 = 0%, and 5 = 0%.

D12 Is endoscopic retrograde cholangiopancreatography recommended as a next step to the initial tests for the diagnosis of pancreatic cancer?

*Statement:*

Endoscopic retrograde cholangiopancreatography is especially recommended for the diagnosis of pancreatic duct stenosis, which is difficult to differentiate from inflammatory lesions by other imaging modalities, or of pancreatic duct stenosis, which could be a manifestation of early pancreatic cancer.

Recommendation strength: weak; evidence level: C; agreement rates (*N = *50): 1 = 0%, 2 = 94%, 3 = 4%, 4 = 0%, and 5 = 2%.

D13 Is positron emission tomography (PET) recommended as a diagnostic method in patients with suspected pancreatic cancer?

*Statement:*

Positron emission tomography is not recommended as a diagnostic or qualitative diagnostic tool in patients with suspected pancreatic cancer.

Recommendation strength: weak; evidence level: C; agreement rates (*N = *50): 1 = 0%, 2 = 10%, 3 = 84%, 4 = 2%, and 5 = 4%.

D14 In patients presenting with a mass lesion in the pancreas, is EUS-guided fine-needle aspiration or biopsy recommended as a diagnostic procedure?

*Statement:*

EUS-guided fine-needle aspiration or biopsy is recommended for the histopathological diagnosis of mass lesions in the pancreas.

Recommendation strength: weak; evidence level: B; agreement rates (*N = *47): 1 = 11%, 2 = 87%, 3 = 2%, 4 = 0%, and 5 = 0%.

D15 If patients presenting with a mass lesion of the pancreas, is transabdominal ultrasound-guided biopsy recommended as a diagnostic procedure?

*Statement:*

In patients presenting with a mass lesion in the pancreas, transabdominal ultrasound-guided biopsy is recommended as a diagnostic procedure.

Recommendation strength: weak; evidence level: B; agreement rates (*N = *51): 1 = 2%, 2 = 94%, 3 = 2%, 4 = 0%, and 5 = 2%.

D16 Is needle biopsy recommended for detecting genetic abnormalities in patients with pancreatic cancer?

*Statement:*

Needle biopsy is recommended for the diagnosis of genetic abnormalities in patients with pancreatic cancer.

Recommendation strength: weak; evidence level: C; agreement rates (*N = *51): 1 = 0%, 2 = 98%, 3 = 0%, 4 = 0%, and 5 = 2%.

D17 Is endoscopic retrograde cholangiopancreatography with pancreatic fluid cytology recommended in patients with certain pancreatic duct findings and no mass lesions?

*Statement:*

Endoscopic retrograde cholangiopancreatography with pancreatic fluid cytology is recommended in patients with certain pancreatic duct findings and no mass lesions. However, particular attention should be paid to the possible precipitation of acute pancreatitis by endoscopic retrograde cholangiopancreatography.

Recommendation strength: weak; evidence level: D; agreement rates (*N = *49): 1 = 0%, 2 = 100%, 3 = 0%, 4 = 0%, and 5 = 0%.

D18 Is abdominal MRI recommended for the staging of pancreatic cancer and for assessment of its resectability?

*Statement:*

Contrast-enhanced MRI is recommended for pancreatic cancer staging and for assessment of its resectability, especially for the detection of liver metastasis.

Recommendation strength: weak; evidence level: C; agreement rates (*N = *51): 1 = 0%, 2 = 98%, 3 = 2%, 4 = 0%, and 5 = 0%.

D19 Is EUS recommended for the staging of pancreatic cancer and for assessment of its resectability?

*Statement:*

When contrast-enhanced CT cannot definitively determine the disease stage/resectability, addition of EUS is recommended, because EUS is superior to contrast-enhanced CT for diagnosing T-factor/N-factor/vascular invasion.

Recommendation strength: weak; evidence level: B; agreement rates (*N = *51): 1 = 2%, 2 = 96%, 3 = 2%, 4 = 0%, and 5 = 0%.

D20 Is PET recommended for the staging of pancreatic cancer and for assessment of its resectability?

*Statement:*

Positron emission tomography is recommended in patients with suspected distant metastasis, as PET is more specific than CT for the diagnosis of distant metastasis.

Recommendation strength: weak; evidence level: C; agreement rates (*N = *50): 1 = 2%, 2 = 92%, 3 = 6%, 4 = 0%, and 5 = 0%

D21 Is laparoscopy recommended for evaluating the disease stage in pancreatic cancer patients with suspected distant metastasis?

*Statement:*

Laparoscopy is recommended for evaluating the disease stage in pancreatic cancer patients, because it is useful for identifying small metastases on the surface of the liver and peritoneal metastases.

Recommendation strength: weak; evidence level: C; agreement rates (*N = *51): 1 = 2%, 2 = 94%, 3 = 4%, 4 = 0%, and 5 = 0%.

D22 Is preoperative assessment of nutrition and body composition (muscle and fat mass) along with blood biochemistry recommended in patients with pancreatic cancer?

*Statement:*

Assessment of the preoperative nutritional status and body composition is recommended in patients with pancreatic cancer, because these variables have been shown to contribute to prediction of the long-term prognosis and postoperative complications in patients undergoing surgery for pancreatic cancer.

Recommendation strength: weak; evidence level: D; agreement rates (*N = *49): 1 = 8%, 2 = 90%, 3 = 2%, 4 = 0%, and 5 = 0%.

## Treatment

### Treatment of resectable disease or R

#### Operation or O

RO1 Is surgical treatment for pancreatic cancer recommended at a facility with a large volume of surgical cases?

*Statement:*

Surgical treatment for pancreatic cancer is recommended at high-volume facilities.

Recommendation strength: weak; evidence level: B; agreement rates (*N = *51): 1 = 10%, 2 = 88%, 3 = 2%, 4 = 0%, and 5 = 0%.

RO2 Is surgical treatment recommended for pancreatic cancer patients with positive peritoneal lavage cytology?

*Statement:*

Surgical treatment is not recommended as the first-line therapy in pancreatic cancer patients with a positive peritoneal lavage cytology.

Recommendation strength: weak; evidence level: C; agreement rates (*N = *50): 1 = 2%, 2 = 8%, 3 = 80%, 4 = 6%, and 5 = 4%.

RO3 Is combined portal vein resection recommended in patients with pancreatic cancer?

*Statement:*

It is not yet clear whether combined portal vein resection for pancreatic cancer might improve the prognosis in patients with pancreatic cancer. However, when curative resection is expected, combined portal vein resection is recommended.

Recommendation strength: weak; evidence level: D; agreement rates (*N = *51): 1 = 6%, 2 = 88%, 3 = 2%, 4 = 2%, and 5 = 2%.

RO4 Is prophylactic extended lymph node and nerve plexus dissection recommended in patients with pancreatic cancer?

*Statement:*

Prophylactic extended lymph node and nerve plexus dissection is not recommended in patients with pancreatic cancer, because it has not been shown to contribute to prolong survival.

Recommendation strength: strong; evidence level: A; agreement rates (*N = *50): 1 = 0%, 2 = 4%, 3 = 22%, 4 = 74%, and 5 = 0%.

RO5 Is minimally invasive pancreaticoduodenectomy recommended in patients with invasive ductal carcinoma who are candidates for pancreaticoduodenectomy?

*Statement:*

Minimally invasive pancreaticoduodenectomy is recommended in patients with invasive ductal carcinoma who are candidates for pancreaticoduodenectomy, but only at facilities specializing in the treatment of pancreatic cancer.

Recommendation strength: weak; evidence level: C; agreement rates (*N = *51): 1 = 2%, 2 = 86%, 3 = 2%, 4 = 0%, and 5 = 10%.

RO6 Is minimally invasive distal pancreatectomy recommended in patients with invasive ductal carcinoma who are candidates for distal pancreatectomy?

*Statement:*

1. Laparoscopic distal pancreatectomy is recommended in patients with invasive ductal carcinoma who are candidates for distal pancreatectomy, but only at facilities specializing in the treatment of pancreatic cancer.

Recommendation strength: weak; evidence level: C; agreement rates (*N = *50): 1 = 2%, 2 = 94%, 3 = 0%, 4 = 0%, and 5 = 4%.

2. Robot-assisted distal pancreatectomy is recommended in patients with invasive ductal carcinoma who are candidates for distal pancreatectomy, but only at facilities specializing in the treatment of pancreatic cancer.

Recommendation strength: weak; evidence level: C; agreement rates (*N = *49): 1 = 2%, 2 = 92%, 3 = 2%, 4 = 0%, and 5 = 4%.

RO7 Is long-term regular surveillance recommended after surgical resection for pancreatic cancer?

*Statement:*

Continued regular long-term surveillance of pancreatic cancer patients is recommended, even in patients surviving for more than 5 years after resection.

Recommendation strength: weak; evidence level: D; agreement rates (*N = *51): 1 = 4%, 2 = 94%, 3 = 2%, 4 = 0%, and 5 = 0%.

RO8 Is perioperative pancreatic enzyme replacement therapy recommended in pancreatic cancer patients?

*Statement:*

Perioperative pancreatic enzyme replacement therapy is recommended in pancreatic cancer patients with suspected/confirmed pancreatic exocrine insufficiency.

Recommendation strength: weak; evidence level: C; agreement rates (*N = *51): 1 = 2%, 2 = 98%, 3 = 0%, 4 = 0%, and 5 = 0%.

RO9 Is surgical treatment recommended for elderly pancreatic cancer patients who are 80 years of age or older?

*Statement:*

Surgical treatment is recommended even in elderly patients with pancreatic cancer who are 80 years of age or older, when curative resection is expected.

Recommendation strength: weak; evidence level: C; agreement rates (*N = *51): 1 = 0%, 2 = 98%, 3 = 2%, 4 = 0%, and 5 = 0%.

RO10 Is total pancreatectomy recommended to achieve curative resection in patients with pancreatic cancer?

*Statement:*

Total pancreatectomy is recommended to achieve curative resection in patients with pancreatic cancer.

Recommendation strength: weak; evidence level: C; agreement rates (*N = *51): 1 = 0%, 2 = 98%, 3 = 0%, 4 = 2%, and 5 = 0%.

### Adjuvant or A

RA1 Is neoadjuvant therapy recommended for patients with resectable pancreatic cancer?

*Statement:*

Combined gemcitabine + S-1 therapy is recommended as preoperative neoadjuvant therapy in patients with resectable pancreatic cancer.

Recommendation strength: weak; evidence level: C; agreement rates (*N = *49): 1 = 14%, 2 = 86%, 3 = 0%, 4 = 0%, and 5 = 0%.

RA2 Is adjuvant chemotherapy recommended in patients with pancreatic cancer?

*Statement:*

1. Postoperative adjuvant chemotherapy is recommended in patients with pancreatic cancer who have received macroscopic radical resection.

Recommendation strength: strong; evidence level: A; agreement rates (*N = *50): 1 = 82%, 2 = 18%, 3 = 0%, 4 = 0%, and 5 = 0%.

2. S-1 monotherapy is recommended as an adjuvant chemotherapeutic agent in patients with pancreatic cancer.

Recommendation strength: strong; evidence level: A; agreement rates (*N = *47): 1 = 87%, 2 = 13%, 3 = 0%, 4 = 0%, and 5 = 0%.

3. Gemcitabine hydrochloride monotherapy is recommended for patients showing poor tolerance to S-1.

Recommendation strength: strong; evidence level: A; agreement rates (*N = *47): 1 = 74%, 2 = 23%, 3 = 2%, 4 = 0%, and 5 = 0%.

4. Combined gemcitabine hydrochloride + capecitabine therapy (not covered by health insurance) and modified fluorouracil, leucovorin, irinotecan, and oxaliplatin (modified FOLFIRINOX) therapy (not covered by health insurance) are recommended for patients with pancreatic cancer, based on the results of phase III studies conducted outside Japan.

Recommendation strength: weak; evidence level: A; agreement rates (*N = *47): 1 = 4%, 2 = 87%, 3 = 4%, 4 = 0%, and 5 = 4%.

## Treatment of borderline resectable disease or B

### Operation or O

BO1 Is surgical treatment recommended for patients with borderline resectable pancreatic cancer?

*Statement:*

In patients with borderline resectable pancreatic cancer, it is recommended that reassessment be performed prior to surgery to determine if the cancer can be curatively resected, by evaluating the therapeutic efficacy of preoperative neoadjuvant therapy.

Recommendation strength: weak; evidence level: C; agreement rates (*N = *51): 1 = 2%, 2 = 94%, 3 = 4%, 4 = 0%, and 5 = 0%.

BO2 Is combined arterial resection recommended in patients with pancreatic cancer?

*Statement:*

1. Distal pancreatectomy with celiac artery resection is recommended.

Recommendation strength: weak; evidence level: C; agreement rates (N = 50): 1 = 0%, 2 = 96%, 3 = 2%, 4 = 2%, and 5 = 0%.

2. Combined resection of the hepatic artery is recommended.

Recommendation strength: weak; evidence level: C; agreement rates (N = 49): 1 = 0%, 2 = 100%, 3 = 0%, 4 = 0%, and 5 = 0%.

3. Combined resection of the superior mesenteric artery is not recommended.

Recommendation strength: weak; evidence level: C; agreement rates (N = 49): 1 = 0%, 2 = 0%, 3 = 90%, 4 = 8%, and 5 = 2%.

#### Adjuvant or A

BA1 What is the recommended preoperative neoadjuvant therapy for borderline resectable pancreatic cancer?

*Statement:*

As neoadjuvant therapy for borderline resectable pancreatic cancer,

1. Chemoradiotherapy is recommended.

Recommendation strength: weak; evidence level: C; agreement rates (*N = *44): 1 = 2%, 2 = 93%, 3 = 2%, 4 = 0%, and 5 = 2%.

2. Chemotherapy alone is recommended.

Recommendation strength: weak; evidence level: C; agreement rates (*N = *44): 1 = 2%, 2 = 98%, 3 = 0%, 4 = 0%, and 5 = 0%.

BA2 Is postoperative adjuvant chemotherapy recommended for patients with borderline resectable pancreatic cancer?

*Statement:*

Postoperative adjuvant chemotherapy is recommended for patients with borderline resectable pancreatic cancer.

Recommendation strength: weak; evidence level: C; agreement rates (*N = *49): 1 = 8%, 2 = 92%, 3 = 0%, 4 = 0%, and 5 = 0%.

#### Treatment of locally advanced disease or L

L1 What is the first-line treatment recommended for patients with locally advanced, unresectable pancreatic cancer?

*Statement:*

1. Chemoradiotherapy is recommended.

Recommendation strength: weak; evidence level: B; agreement rates (*N = *48: 1 = 4%, 2 = 96%, 3 = 0%, 4 = 0%, and 5 = 0%.

2. Chemotherapy alone is recommended.

Recommendation strength: weak; evidence level: B; agreement rates (*N = *48): 1 = 6%, 2 = 94%, 3 = 0%, 4 = 0%, and 5 = 0%.

## Radiation or R

LR1 What is the chemoradiotherapy regimen recommended for patients with locally advanced, unresectable pancreatic cancer?

*Statement:*

1. For patients with locally advanced, unresectable pancreatic cancer scheduled to receive chemoradiotherapy, concurrent use of fluoropyrimidine with radiotherapy is recommended.

Recommendation strength: weak; evidence level: C; agreement rates (*N = *47): 1 = 4%, 2 = 96%, 3 = 0%, 4 = 0%, and 5 = 0%.

2. For patients with locally advanced, unresectable pancreatic cancer scheduled to receive chemoradiotherapy, concurrent use of gemcitabine hydrochloride with radiotherapy is recommended.

Recommendation strength: weak; evidence level: C; agreement rates (*N = *47): 1 = 4%, 2 = 94%, 3 = 0%, 4 = 0%, and 5 = 2%.

LR2 Is elective nodal irradiation for regional lymph nodes recommended in radiotherapy in patients with locally advanced, unresectable pancreatic cancer?

*Statement:*

In patients with locally advanced, unresectable pancreatic cancer scheduled to receive radiotherapy, elective nodal irradiation for the para-aortic lymph nodes is not recommended.

Recommendation strength: weak; evidence level: D; agreement rates (*N = *49): 1 = 0%, 2 = 14%, 3 = 84%, 4 = 0%, and 5 = 2%.

LR3 Is induction chemotherapy recommended prior to chemoradiotherapy in patients with locally advanced, unresectable pancreatic cancer?

*Statement:*

Gemcitabine hydrochloride monotherapy is not recommended as induction chemotherapy prior to chemoradiotherapy in patients with locally advanced, unresectable pancreatic cancer.

Recommendation strength: weak; evidence level: C; agreement rates (*N = *47): 1 = 0%, 2 = 6%, 3 = 89%, 4 = 0%, and 5 = 4%.

LR4 For unresectable pancreatic cancer patients with pain, is radiotherapy alone or chemoradiotherapy recommended for the primary lesion?

*Statement:*

Radiotherapy alone or chemoradiotherapy for the primary lesion is recommended for unresectable pancreatic cancer patients with pain.

Recommendation strength: weak; evidence level: C; agreement rates (*N = *50): 1 = 0%, 2 = 100%, 3 = 0%, 4 = 0%, and 5 = 0%.

LR5 Is high-precision radiotherapy (intensity-modulated radiation therapy, stereotactic body radiotherapy, and particle beam therapy) recommended as radiation therapy in patients with locally advanced, unresectable pancreatic cancer?

*Statement:*

Increased radiation dose using high-precision radiotherapy is recommended in patients with locally advanced, unresectable pancreatic cancer.

Recommendation strength: weak; evidence level: C; agreement rates (*N = *50): 1 = 0%, 2 = 98%, 3 = 0%, 4 = 0%, and 5 = 2%.

LR6 Is hyperthermia recommended in combination with chemoradiotherapy in patients with locally advanced, unresectable pancreatic cancer?

*Statement:*

It is not possible to make a clear recommendation at this time about the need for inducing hyperthermia in combination with chemoradiotherapy in patients with locally advanced, unresectable pancreatic cancer.

Recommendation strength: no recommendation; evidence level: D; agreement rates (*N = *49): 1 = 0%, 2 = 2%, 3 = 6%, 4 = 0%, and 5 = 92%.

### Chemotherapy or C

LC1 What is the first-line chemotherapy recommended for patients with locally advanced, unresectable pancreatic cancer?

*Statement:*

3. Combined fluorouracil, leucovorin, irinotecan, and oxaliplatin (FOLFIRINOX) therapy is recommended.

Recommendation strength: weak; evidence level: C; agreement rates (*N = *43): 1 = 19%, 2 = 81%, 3 = 0%, 4 = 0%, and 5 = 0%.

4. Combined gemcitabine hydrochloride + nab-paclitaxel therapy is recommended.

Recommendation strength: weak; evidence level: C; agreement rates (*N = *43): 1 = 23%, 2 = 77%, 3 = 0%, 4 = 0%, and 5 = 0%.

5. Gemcitabine hydrochloride monotherapy is recommended.

Recommendation strength: weak; evidence level: C; agreement rates (*N = *43): 1 = 2%, 2 = 93%, 3 = 2%, 4 = 0%, and 5 = 2%.

6. S-1 monotherapy is recommended.

Recommendation strength: weak; evidence level: C; agreement rates (*N = *43): 1 = 5%, 2 = 91%, 3 = 5%, 4 = 0%, and 5 = 0%.

LC2 (MC2) Is second-line chemotherapy recommended for patients with unresectable pancreatic cancer?

*Statement:*

7. Second-line chemotherapy is recommended for unresectable pancreatic cancer patients who are refractory to first-line therapy.

Recommendation strength: weak; evidence level: B; agreement rates (*N = *44): 1 = 68%, 2 = 32%, 3 = 0%, 4 = 0%, and 5 = 0%.

8. Combined fluorouracil + calcium folinate + nanoliposomal irinotecan is recommended after first-line treatment with a gemcitabine hydrochloride-containing regimen.

Recommendation strength: weak; evidence level: B; agreement rates (*N = *44): 1 = 55%, 2 = 45%, 3 = 0%, 4 = 0%, and 5 = 0%.

9. Use of a fluorouracil-containing regimen (including FOLFIRINOX therapy and S-1 monotherapy) is recommended after first-line therapy with a gemcitabine hydrochloride-containing regimen.

Recommendation strength: weak; evidence level: C; agreement rates (*N = *44): 1 = 5%, 2 = 95%, 3 = 0%, 4 = 0%, and 5 = 0%.

10. Use of a gemcitabine hydrochloride-containing regimen is recommended after first-line treatment with a fluorouracil-containing regimen.

Recommendation strength: weak; evidence level: C; agreement rates (*N = *44): 1 = 2%, 2 = 98%, 3 = 0%, 4 = 0%, and 5 = 0%.

11. Pembrolizumab monotherapy is recommended for microsatellite instability-high cases.

Recommendation strength: weak; evidence level: C; agreement rates (*N = *44): 1 = 0%, 2 = 100%, 3 = 0%, 4 = 0%, and 5 = 0%.

12. Pembrolizumab monotherapy is recommended for tumor mutational burden-high cases.

Recommendation strength: weak; evidence level: C; agreement rates (*N = *44): 1 = 0%, 2 = 95%, 3 = 0%, 4 = 0%, and 5 = 5%.

13. Entrectinib monotherapy or larotrectinib monotherapy is recommended for cases with tumors harboring NTRK gene fusions.

Recommendation strength: weak; evidence level: C; agreement rates (*N = *44): 1 = 0%, 2 = 98%, 3 = 0%, 4 = 0%, and 5 = 2%.

LC3 (MC3) What is the first-line chemotherapy recommended for elderly patients with advanced pancreatic cancer?

*Statement:*

As first-line chemotherapy for elderly patients with advanced pancreatic cancer in consideration of the performance status and comorbidities,

1. Combined gemcitabine hydrochloride + nab-paclitaxel therapy is recommended.

Recommendation strength: weak; evidence level: C; agreement rates (*N = *42): 1 = 0%, 2 = 100%, 3 = 0%, 4 = 0%, and 5 = 0%.

2. Gemcitabine hydrochloride monotherapy is recommended

Recommendation strength: weak; evidence level: C; agreement rates (*N = *42): 1 = 0%, 2 = 98%, 3 = 0%, 4 = 0%, and 5 = 2%.

3. S-1 monotherapy is recommended.

Recommendation strength: weak; evidence level: C; agreement rates (*N = *42): 1 = 0%, 2 = 100%, 3 = 0%, 4 = 0%, and 5 = 0%.

LC4 (MC4) What is the chemotherapy regimen recommended for pancreatic cancer patients with a germline pathogenic variant of BRCA1/2?

Statement

1. Use of a platinum-containing regimen is recommended for pancreatic cancer patients with pathological germline variants of BRCA1/2.

Recommendation strength: weak; evidence level: C; agreement rates (*N = *47): 1 = 4%, 2 = 96%, 3 = 0%, 4 = 0%, and 5 = 0%.

2. Maintenance therapy with olaparib is recommended as one of the treatment options for pancreatic cancer patients with distant metastases in whom disease progression has been suppressed for a certain period of time with a platinum-containing regimen.

Recommendation strength: weak; evidence level: C; agreement rates (*N = *47): 1 = 4%, 2 = 94%, 3 = 0%, 4 = 0%, and 5 = 2%.

3. Maintenance therapy with olaparib is recommended as one of the treatment options for patients with locally advanced pancreatic cancer in whom disease progression has been suppressed for a certain period of time with a platinum-containing regimen.

Recommendation strength: weak; evidence level: C; agreement rates (*N = *47): 1 = 4%, 2 = 96%, 3 = 0%, 4 = 0%, and 5 = 0%

### Operation or O

LO1 Is resection of the primary lesion after multidisciplinary treatment recommended in patients with locally advanced cancer who were judged as being unsuitable candidates for resection at the first examination?

*Statement:*

Resection of the primary lesion after multidisciplinary treatment is recommended as one of the treatment options for patients with locally advanced pancreatic cancer in whom the disease was judged as being unresectable at the first examination, if the treatment is successful and the cancer becomes resectable.

Recommendation strength: weak; evidence level: C; agreement rates (*N = *50): 1 = 0%, 2 = 96%, 3 = 2%, 4 = 0%, and 5 = 2%.

### Adjuvant or A

LA1 Is adjuvant chemotherapy recommended after primary lesion resection for patients with locally advanced, unresectable pancreatic cancer at the time of the initial visit?

*Statement:*

Adjuvant chemotherapy after primary lesion resection is recommended for patients with locally advanced, unresectable pancreatic cancer at the time of initial examination.

Recommendation strength: weak; evidence level: D; agreement rates (*N = *48): 1 = 4%, 2 = 94%, 3 = 0%, 4 = 0%, and 5 = 2%.

## Treatment of metastatic disease or M

### Chemotherapy or C

MC1 What is the first-line chemotherapy recommended for pancreatic cancer patients with distant metastases?

*Statement:*

As first-line chemotherapy for pancreatic cancer patients with distant metastases,

14. FOLFIRINOX therapy is recommended.

Recommendation strength: strong; evidence level: A; agreement rates (*N = *43): 1 = 91%, 2 = 9%, 3 = 0%, 4 = 0%, and 5 = 0%.

15. Combined gemcitabine hydrochloride + nab-paclitaxel therapy is recommended.

Recommendation strength: strong; evidence level: A; agreement rates (*N = *43): 1 = 91%, 2 = 9%, 3 = 0%, 4 = 0%, and 5 = 0%.

For patients in whom the above treatments are not indicated because of the general condition, age, or other reasons.

16. Gemcitabine hydrochloride monotherapy is recommended.

Recommendation strength: weak; evidence level: A; agreement rates (*N = *43): 1 = 5%, 2 = 93%, 3 = 2%, 4 = 0%, and 5 = 0%.

17. S-1 monotherapy is recommended.

Recommendation strength: weak; evidence level: A; agreement rates (*N = *43): 1 = 2%, 2 = 98%, 3 = 0%, 4 = 0%, and 5 = 0%.

MC2 (LC2) Is second-line chemotherapy recommended for patients with unresectable pancreatic cancer?

*Statement:*

18. Second-line chemotherapy is recommended for patients with unresectable pancreatic cancer who are refractory to first-line therapy.

Recommendation strength: strong; evidence level: B; agreement rates (*N = *44): 1 = 68%, 2 = 32%, 3 = 0%, 4 = 0%, and 5 = 0%.

19. Combined fluorouracil + calcium folinate + nanoliposomal irinotecan is recommended after first-line treatment with a gemcitabine hydrochloride-containing regimen.

Recommendation strength: weak; evidence level: B; agreement rates (*N = *44): 1 = 55%, 2 = 45%, 3 = 0%, 4 = 0%, and 5 = 0%.

20. Use of a fluorouracil-containing regimen (including FOLFIRINOX therapy and S-1 monotherapy) is recommended after first-line treatment with a gemcitabine hydrochloride-containing regimen.

Recommendation strength: weak; evidence level: C; agreement rates (*N = *44): 1 = 5%, 2 = 95%, 3 = 0%, 4 = 0%, and 5 = 0%.

21. Use of a gemcitabine hydrochloride-containing regimen is recommended after first-line treatment with a fluorouracil-containing regimen.

Recommendation strength: weak; evidence level: C; agreement rates (*N = *44): 1 = 2%, 2 = 98%, 3 = 0%, 4 = 0%, and 5 = 0%.

22. Pembrolizumab monotherapy is recommended for microsatellite instability-high cases.

Recommendation strength: weak; evidence level: C; agreement rates (*N = *44): 1 = 0%, 2 = 100%, 3 = 0%, 4 = 0%, and 5 = 0%.

23. Pembrolizumab monotherapy is recommended for tumor mutational burden-high cases.

Recommendation strength: weak; evidence level: C; agreement rates (*N = *44): 1 = 0%, 2 = 95%, 3 = 0%, 4 = 0%, and 5 = 5%.

24. Entrectinib monotherapy or larotrectinib monotherapy is recommended for cases with tumors harboring NTRK gene fusions.

Recommendation strength: weak; evidence level: C; agreement rates (*N = *44): 1 = 0%, 2 = 98%, 3 = 0%, 4 = 0%, and 5 = 2%.

MC3 (LC3) What is the first-line chemotherapy recommended in elderly patients with advanced pancreatic cancer?

*Statement:*

As first-line chemotherapy in elderly patients with advanced pancreatic cancer, in consideration of the performance status and comorbidities,

1. Combined gemcitabine hydrochloride + nab-paclitaxel therapy is recommended.

Recommendation strength: weak; evidence level: C; agreement rates (*N = *42): 1 = 0%, 2 = 100%, 3 = 0%, 4 = 0%, and 5 = 0%.

2. Gemcitabine hydrochloride monotherapy is recommended.

Recommendation strength: weak; evidence level: C; agreement rates (*N = *42): 1 = 0%, 2 = 98%, 3 = 0%, 4 = 0%, and 5 = 2%.

3. S-1 monotherapy is recommended.

Recommendation strength: weak; evidence level: C; agreement rates (*N = *42): 1 = 0%, 2 = 100%, 3 = 0%, 4 = 0%, and 5 = 0%.

MC4 (LC4) What is the recommended chemotherapy for pancreatic cancer patients with germline pathogenic variants of BRCA1/2?

*Statement:*

1. Use of a platinum-containing regimen is recommended for pancreatic cancer patients with pathological germline variants of BRCA1/2.

Recommendation strength: weak; evidence level: C; agreement rates (*N = *47): 1 = 4%, 2 = 96%, 3 = 0%, 4 = 0%, and 5 = 0%.

2. Maintenance therapy with olaparib is recommended as one of the treatment options for pancreatic cancer patients with distant metastases in whom disease progression has remained suppressed for a certain period of time with a platinum-containing regimen.

Recommendation strength: weak; evidence level: C; agreement rates (*N = *47): 1 = 4%, 2 = 94%, 3 = 0%, 4 = 0%, and 5 = 2%.

3. Maintain therapy with olaparib is recommended as one of the treatment options for patients with locally advanced pancreatic cancer in whom disease progression has remained suppressed for a certain period of time with a platinum-containing regimen.

Recommendation strength: weak; evidence level: C; agreement rates (*N = *47): 1 = 4%, 2 = 96%, 3 = 0%, 4 = 0%, and 5 = 0%.

### Operation or O

MO1Is surgical resection recommended for pancreatic cancer patients with postoperative metastases/recurrences?

*Statement:*

25. Surgical resection of the remnant pancreas is recommended in patients with postoperative metastases/recurrences.

Recommendation strength: weak, evidence level: D, agreement rates (*N = *50): 1 = 0%, 2 = 92%, 3 = 4%, 4 = 0%, and 5 = 4%.

26. Surgical resection is recommended for lung metastases after carefully confirming the surgical indication.

Recommendation strength: weak, evidence level: D, agreement rates (*N = *50): 1 = 0%, 2 = 90%, 3 = 8%, 4 = 0%, and 5 = 2%.

27. Surgical resection is not recommended for metastases other than lung metastases (e.g., liver metastases).

Recommendation strength: weak, evidence level: D, agreement rates (*N = *47): 1 = 0%, 2 = 4%, 3 = 94%, 4 = 2%, and 5 = 0%.

MO2 Is surgical treatment after multidisciplinary treatment recommended for distant metastases that are unresectable at the time of first diagnosis in advanced pancreatic cancer patients?

*Statement:*

In pancreatic cancer patients with distant metastasis that cannot be resected at the time of first diagnosis, it is not clear whether surgical treatment should be performed, even if response to multidisciplinary treatment is obtained at the primary site and/or distant metastasis.

Recommendation strength: no recommendation, evidence level: D, agreement rates (*N = *50): 1 = 0%, 2 = 6%, 3 = 8%, 4 = 0%, and 5 = 86%.

### Radiation or R

MR1 Is radiotherapy recommended for the management of painful bone metastases in patients with advanced pancreatic cancer?

*Statement:*

Radiotherapy is recommended for the management of painful bone metastases in patients with advanced pancreatic cancer.

Recommendation strength: strong; evidence level: B; agreement rates (*N = *50): 1 = 84%, 2 = 16%, 3 = 0%, 4 = 0%, and 5 = 0%.

MR2 Is radiotherapy recommended for pancreatic cancer patients with postoperative metastases/recurrences?

*Statement:*

28. Radiotherapy is recommended for the management of local recurrences and regional lymph-node metastases.

Recommendation strength: weak; evidence level: D; agreement rates (*N = *50): 1 = 2%, 2 = 92%, 3 = 2%, 4 = 0%, and 5 = 4%.

29. Radiotherapy is recommended for the management of lung metastases.

Recommendation strength: weak; evidence level: D; agreement rates (*N = *50): 1 = 2%, 2 = 94%, 3 = 2%, 4 = 0%, and 5 = 2%.

30. Radiotherapy is not recommended for the management of liver metastases.

Recommendation strength: weak; evidence level: D; agreement rates (*N = *50): 1 = 0%, 2 = 12%, 3 = 84%, 4 = 2%, and 5 = 2%.

## Supportive and palliative medicine or S

Stenting or St

SSt1 Is the endoscopic transpapillary route recommended for biliary drainage in patients with unresectable pancreatic cancer?

*Statement:*

The endoscopic transpapillary route is recommended for biliary drainage in patients with unresectable pancreatic cancer.

Recommendation strength: weak; evidence level: B; agreement rates (*N = *45): 1 = 9%, 2 = 91%, 3 = 0%, 4 = 0%, and 5 = 0%.

SSt2 Which of the two types of stents—plastic stents or metallic stents—recommended in resectable or borderline resectable pancreatic cancer patients with obstructive jaundice?

*Statement:*

31. Use of metallic stents is recommended in resectable or borderline resectable pancreatic cancer patients with obstructive jaundice.

Recommendation strength: weak; evidence level: C; agreement rates (*N = *45): 1 = 13%, 2 = 82%, 3 = 4%, 4 = 0%, and 5 = 0%.

32. When the waiting period for surgery is short, use of a plastic stent is recommended.

Recommendation strength: weak; evidence level: C; agreement rates (*N = *34): 1 = 0%, 2 = 82%, 3 = 15%, 4 = 0%, and 5 = 3%.

SSt3 Is the use of covered metallic stents recommended in unresectable pancreatic cancer patients with obstructive jaundice?

*Statement:*

Use of a covered metallic stent is recommended in unresectable pancreatic cancer patients with obstructive jaundice.

Recommendation strength: weak; evidence level: B; agreement rates (*N = *43): 1 = 12%, 2 = 88%, 3 = 0%, 4 = 0%, and 5 = 0%

SSt4 Is endoscopic gastrointestinal stent insertion recommended for unresectable pancreatic cancer with gastrointestinal obstruction?

*Statement:*

Endoscopic gastrointestinal stent insertion is recommended for unresectable pancreatic cancer patients with gastrointestinal obstruction. In patients with a prolonged life expectancy, surgical gastro-jejunal anastomosis is recommended.

Recommendation strength: weak; evidence level: C; agreement rates (*N = *45): 1 = 4%, 2 = 96%, 3 = 0%, 4 = 0%, and 5 = 0%.

SSt5 Is the endoscopic transgastrointestinal route for biliary drainage recommended for managing obstructive jaundice in patients with pancreatic cancer complicated by gastric and duodenal obstruction?

*Statement:*

The endoscopic transgastrointestinal route for biliary drainage is recommended in facilities with personnel skilled in the procedure for managing obstructive jaundice in patients with pancreatic cancer complicated by gastric and duodenal obstruction.

Recommendation strength: weak; evidence level: D; agreement rates (*N = *40): 1 = 10%, 2 = 88%, 3 = 0%, 4 = 0%, and 5 = 3%.

SSt6 Is the use of metallic stents during chemotherapy and/or radiation therapy recommended in unresectable pancreatic cancer patients with obstructive jaundice?

*Statement:*

1. Use of metallic stents is recommended in pancreatic cancer patients with obstructive jaundice receiving chemotherapy.

Recommendation strength: weak; evidence level: D; agreement rates (*N = *40): 1 = 8%, 2 = 80%, 3 = 3%, 4 = 0%, and 5 = 10%.

2. No clear recommendation can be made at this time for the use of metallic stents in pancreatic cancer patients with obstructive jaundice receiving radiation therapy.

Recommendation strength: no recommendation; evidence level: D; agreement rates (*N = *36): 1 = 0%, 2 = 14%, 3 = 11, 4 = 0%, and 5 = 75%.

### Supportive & palliative medicine or Sp

SSp1 Are interventions directed at reducing the psychological distress recommended for patients with pancreatic cancer and their families?

*Statement:*

Provision of systematic support by a multidisciplinary team consisting of multiple experts, such as a palliative care team, is recommended even from the early stages of treatment for patients with advanced pancreatic cancer and their families.

Recommendation strength: weak; evidence level: D; agreement rates (*N = *48): 1 = 4%, 2 = 96%, 3 = 0%, 4 = 0%, and 5 = 0%.

SSp2 Are non-opioid analgesics, opioid analgesics, nerve blocks, and adjuvant analgesics recommended for pancreatic cancer patients with cancer pain?

*Statement:*

33. Pain treatment with a non-opioid/opioid analgesic(s) is recommended for pancreatic cancer patients with cancer pain.

Recommendation strength: weak; evidence level: C; agreement rates (*N = *48): 1 = 63%, 2 = 38%, 3 = 0%, 4 = 0%, and 5 = 0%.

1. Nerve blocks are recommended for pancreatic cancer patients with cancer pain.

Recommendation strength: weak; evidence level: B; agreement rates (*N = *48): 1 = 0%, 2 = 92%, 3 = 6%, 4 = 0%, and 5 = 2%.

1. Adjuvant analgesics are not recommended for pancreatic cancer patients with cancer pain.

Recommendation strength: weak; evidence level: D; agreement rates (*N = *48): 1 = 6%, 2 = 10%, 3 = 67%, 4 = 2%, and 5 = 15%.

SSp3 Is exercise therapy recommended for patients with pancreatic cancer?

*Statement:*

Exercise therapy is recommended for patients with pancreatic cancer.

Recommendation strength: weak; evidence level: C; agreement rates (*N = *49): 1 = 0%, 2 = 94%, 3 = 0%, 4 = 0%, and 5 = 6%.

SSp4 Is rehabilitation treatment, including exercise therapy, before surgery recommended for pancreatic cancer patients scheduled to undergo surgery?

*Statement:*

Rehabilitation treatment, including exercise therapy, before surgery is recommended for pancreatic cancer patients scheduled to undergo surgery.

Recommendation strength: weak; evidence level: C; agreement rates (*N = *49): 1 = 0%, 2 = 88%, 3 = 0%, 4 = 0%, and 5 = 12%.

SSp5 Is advance care planning recommended for patients with advanced pancreatic cancer?

*Statement:*

Advance care planning is recommended for patients with advanced pancreatic cancer.

Recommendation strength: weak; evidence level: C; agreement rates (*N = *49): 1 = 0%, 2 = 100%, 3 = 0%, 4 = 0%, and 5 = 0%.

SSp6 Are pregabalin, duloxetine, and mirogabalin recommended for the management of peripheral neuropathy caused by FOLFIRINOX therapy or combined gemcitabine hydrochloride + nab-paclitaxel therapy in patients with pancreatic cancer?

*Statement:*

1. Use of duloxetine is recommended for managing peripheral neuropathy associated with FOLFIRINOX therapy or combined gemcitabine hydrochloride + nab-paclitaxel therapy in patients with pancreatic cancer.

Recommendation strength: weak; evidence level: C; agreement rates (*N = *48): 1 = 0%, 2 = 100%, 3 = 0%, 4 = 0%, and 5 = 0%.

2. Use of pregabalin is recommended for managing peripheral neuropathy associated with FOLFIRINOX therapy or combined gemcitabine hydrochloride + nab-paclitaxel therapy in patients with pancreatic cancer.

Recommendation strength: weak; evidence level: C; agreement rates (*N = *48): 1 = 0%, 2 = 96%, 3 = 0%, 4 = 0%, and 5 = 4%.

3. Use of mirogabalin may be considered for managing peripheral neuropathy associated with FOLFIRINOX therapy or combined gemcitabine hydrochloride + nab-paclitaxel therapy in patients with pancreatic cancer.

Recommendation strength: weak; evidence level: D; agreement rates (*N = *48): 1 = 0%, 2 = 94%, 3 = 0%, 4 = 0%, and 5 = 6%.

SSp7 Is anticoagulant therapy for preventing venous thromboembolism recommended in patients with unresectable pancreatic cancer undergoing chemotherapy?

*Statement:*

1. Anticoagulant therapy with low-molecular-weight heparin to prevent new onset of venous thromboembolism is recommended in pancreatic cancer patients undergoing chemotherapy (not approved for health insurance coverage in Japan).

Recommendation strength: weak; evidence level: B; agreement rates (*N = *48): 1 = 0%, 2 = 92%, 3 = 4%, 4 = 0%, and 5 = 4%.

1. Anticoagulant therapy with direct-acting oral anticoagulants to prevent new onset of venous thromboembolism is recommended in pancreatic cancer patients undergoing chemotherapy (anticoagulant therapy for preventing venous thromboembolism not approved for health insurance coverage in Japan).

Recommendation strength: weak; evidence level: C; agreement rates (*N = *48): 1 = 0%, 2 = 94%, 3 = 2%, 4 = 0%, and 5 = 4%.

SSp8 Are ghrelin receptor agonists or a combination of nutritional and exercise interventions centered on essential amino acids recommended for cachexia in patients with advanced pancreatic cancer?

*Statement:*

1. Ghrelin receptor agonists are recommended for cachexia in patients with advanced pancreatic cancer.

Recommendation strength: weak; evidence level: D; agreement rates (*N = *44): 1 = 0%, 2 = 100%, 3 = 0%, 4 = 0%, and 5 = 0%.

1. In patients with advanced pancreatic cancer, a clear recommendation cannot be made at this time for using a combination of nutritional and exercise interventions centered on essential amino acids to manage cachexia.

Recommendation strength: no recommendation; evidence level: D; agreement rates (*N = *44): 1 = 0%, 2 = 7%, 3 = 0%, 4 = 0%, and 5 = 93%.

SSp9 Is CART recommended as a treatment for abdominal bloating in advanced pancreatic cancer patients with malignant ascites?

*Statement:*

No clear recommendation for CART can be made at this time for abdominal bloating in advanced pancreatic cancer patients with malignant ascites.

Recommendation strength: no recommendation; evidence level: D; agreement rates (*N = *49): 1 = 0%, 2 = 16%, 3 = 0%, 4 = 0%, and 5 = 84%.

SSp10 Is Communication Technology Training (CST) recommended for physicians expected to participate in important cancer-related discussions?

*Statement:*

Communication skills’ training is recommended for physicians expected to participate in important cancer-related discussions.

Recommendation strength: weak; evidence level: B; agreement rates (*N = *47): 1 = 62%, 2 = 38%, 3 = 0%, 4 = 0%, and 5 = 0%.

## Data Availability

Data sharing is not applicable to this article because this is a synopsis of the guidelines. The original is available from the reference.

## Abbreviations

BR: Borderline resectable
CT: Computed tomography
ERCP: Endoscopic retrograde cholangiopancreatography
EUS: Endoscopic ultrasonography
EUS-FNA: Endoscopic ultrasonography-fine-needle aspiration
JPS: Japan Pancreas Society
PET: Positron emission tomography
US: Ultrasonography
